# The E3 ubiquitin ligase NEDD4 regulates chemoresistance to 5-fluorouracil in colorectal cancer cells by altering JNK signalling

**DOI:** 10.1038/s41419-023-06349-z

**Published:** 2023-12-14

**Authors:** Sushma Anand, Christina Nedeva, Sai V. Chitti, Pamali Fonseka, Taeyoung Kang, Lahiru Gangoda, Nishat I. Tabassum, Suad Abdirahman, Thiruma V. Arumugam, Tracy L. Putoczki, Sharad Kumar, Suresh Mathivanan

**Affiliations:** 1 Department of Biochemistry, La Trobe Institute for Molecular Science, La Trobe University, Melbourne, VIC 3086 Australia; 2 Department of Microbiology, Anatomy, Physiology and Pharmacology, School of Agriculture, Biomedicine and Environment, Centre for Cardiovascular Biology and Disease Research, La Trobe University, Melbourne, Australia; 3 The Walter and Eliza Hall Institute of Medical Research, Melbourne, VIC 3052 Australia; 4 Department of Medical Biology, University of Melbourne, Melbourne, VIC 3052 Australia; 5 Peter MacCallum Cancer Centre, Melbourne, VIC 3052 Australia; 6 Centre for Cancer Biology, University of South Australia, Adelaide, SA 5001 Australia

**Keywords:** Cancer therapeutic resistance, Mechanisms of disease

## Abstract

Colorectal cancer (CRC) is the second leading cause of cancer deaths. Though chemotherapy is the main treatment option for advanced CRC, patients invariably acquire resistance to chemotherapeutic drugs and fail to respond to the therapy. Although understanding the mechanisms regulating chemoresistance has been a focus of intense research to manage this challenge, the pathways governing resistance to drugs are poorly understood. In this study, we provide evidence for the role of ubiquitin ligase NEDD4 in resistance developed against the most commonly used CRC chemotherapeutic drug 5-fluorouracil (5-FU). A marked reduction in NEDD4 protein abundance was observed in a panel of CRC cell lines and patient-derived xenograft samples that were resistant to 5-FU. Knockout of NEDD4 in CRC cells protected them from 5-FU-mediated apoptosis but not oxaliplatin or irinotecan. Furthermore, NEDD4 depletion in CRC cells reduced proliferation, colony-forming abilities and tumour growth in mice. Follow-up biochemical analysis highlighted the inhibition of the JNK signalling pathway in NEDD4-deficient cells. Treatment with the JNK activator hesperidin in NEDD4 knockout cells sensitised the CRC cells against 5-FU. Overall, we show that NEDD4 regulates cell proliferation, colony formation, tumour growth and 5-FU chemoresistance in CRC cells.

## Introduction

Colorectal cancer (CRC) is the third most frequently diagnosed cancer and the second leading cause of cancer deaths globally. The incidence of CRC increases every year in both developed and developing countries due to many lifestyle factors including high cholesterol diet [1]. Currently, chemotherapy is the main treatment option for metastatic CRC and the chemotherapeutic drug 5-fluorouracil (5-FU) is the most commonly used drug for the treatment of CRC patients. To increase the efficacy of treatment, 5-FU is also administered in combination with oxaliplatin and irinotecan [1–4]. However, cancer cells acquire resistance to chemotherapeutic drugs upon prolonged exposure and due to this, treatment failure is common. Hence, resistance to chemotherapeutic drugs, both inherent and acquired, is a major hurdle in the successful treatment of metastatic CRC patients. Therefore, understanding the molecular mechanisms by which CRC cells become resistant to 5-FU-induced apoptosis may provide better avenues to overcome chemoresistance.

Ubiquitination is one of the most well known post-translational modifications that regulate protein turnover, subcellular localisation and overall functionality of proteins [5]. The ubiquitin ligase neural precursor cell-expressed developmentally downregulated 4 (NEDD4) has been attributed to the regulation of invasion, migration, and most importantly resistance to chemotherapeutic drugs in lung adenocarcinoma cells [6]. Similarly, NEDD4 has been shown to contribute to epithelial–mesenchymal transition and cisplatin resistance in nasopharyngeal carcinoma cells [7]. Ubiquitin ligase NEDD4 has also been described both as an oncogene and a tumour suppressor [8–10]. However, the role of NEDD4 in chemoresistance against 5-FU in CRC cells has not been explored.

In this study, the role of NEDD4 in chemoresistance to 5-FU was examined in the context of CRC. In a panel of cell lines and patient-derived xenograft (PDX) samples that are resistant to 5-FU, NEDD4 expression was found to be low. CRISPR/Cas9-based knockout of NEDD4 in wild-type CRC cells protected the cells from 5-FU-induced apoptosis but not from oxaliplatin or irinotecan. Furthermore, knockout of NEDD4 in CRC cells reduced proliferation, colony-forming abilities and tumour growth in mice. Follow-up biochemical analysis highlighted the inhibition of the JNK signalling pathway upon depletion of NEDD4. Treatment of JNK activator hesperidin in NEDD4 knockout cells sensitised the CRC cells against 5-FU. Taken together, these data suggest that NEDD4 regulates cell proliferation, colony formation, tumour growth and chemoresistance to 5-FU in CRC cells.

## Materials and methods

### Cell culture

Human CRC cell line LIM1215 was aquired from the Ludwig Institute for Cancer Research in Melbourne, Australia. The human CRC cell line LIM1215 and *NEDD4* knockout (KO) cells were cultured in RPMI 1640 medium supplemented with 10% FCS (GIBCO, Life Technologies) and 100 U/mL of penicillin–streptomycin at 37 °C in 5% CO_2_.

### Apoptosis assay

Cell death was analysed using the FACS cell cycle-based method. Cells were seeded at a density of 25 × 10^3^ cells per well in a 24-well plate in 1000 µL RPMI 1640 culture medium and allowed to adhere for 2 days. Cells were then treated with 5-FU with indicated concentrations wherever applicable and incubated for 72 h. At this time point, cells were scraped and resuspended. Cell suspension from each well was transferred into a 96-well plate and centrifuged at 300×*g* for 5 min. The supernatant was discarded, and the pellet was resuspended in PI-Hypotonic lysis buffer (0.1% (w/v) sodium citrate, 0.1% Triton X 100 (w/v), 50 μg/mL propidium iodide (Sigma Life Science®) in milliQ and incubated overnight at 4 °C. Samples were then subjected to FACS CANTO II (BD Biosciences) and analysed using FlowJo (TreeStar).

### SDS–PAGE and immunoblotting

Cells were lysed in SDS lysis buffer (1% SDS in 10 mM Tris–HCl pH 7.4). Equal amounts of cell preparations (60 µg) were mixed in 4X SDS sample buffer (8% w/v SDS, 10% v/v glycerol and 0.4% (w/v) bromophenol blue, 200 mM Tris–HCl pH 6.8) and 2 M DTT and boiled at 95 °C for 2 min before loading onto the 12% polyacrylamide gels immersed in 1X Tris–Glycine buffer. Gels were then electrophoresed at a constant voltage of 150 V for 60–90 min. Proteins were transferred onto the nitrocellulose membranes (Thermo Scientific™) using a wet transfer system. Transfer was performed at a constant voltage of 25 V for 2.5 h in transfer buffer (11.5 mM Tris, 95 mM glycine, 20% (v/v) methanol). Following the transfer, membranes were blocked with 10% (w/v) skim milk in TTBS (100 mM Tris–HCl pH 7.5, 150 mM NaCl, 0.05% (v/v) Tween 20) for 1 h at room temperature and washed three times with TTBS (10 min each). Membranes were then incubated with primary antibodies (1:1000). For immunoblotting several antibodies were used: β-actin (4970, Cell Signaling), Cyclin D1 (2978, Cell Signaling), Axin2 (2151, Cell Signaling), pSTAT3 (9134, Cell Signaling), STAT3 (4904, Cell Signaling), pMAPK (9101, Cell Signaling), MAPK (9102, Cell Signaling), pJNK (9251, Cell Signaling), JNK (9252, Cell Signaling), p62 (5114, Cell Signaling), ATG5 (12994, Cell Signaling), E-cadherin (3195, Cell Signaling), YBX1 (4202, Cell Signaling), AKT (9272, Cell Signaling), PI3K (4249, Cell Signaling), p53 (2524, Cell Signaling), Caspase 3 (9662, Cell Signaling), γH2AX (9718, Cell Signaling), Thymidine synthase (9045, Cell Signaling), BCL2 (2876, Cell Signaling), Caspase 8 (ALX-804-242-C100, Enzo), β-catenin (7199, Santa Cruz) and NEDD4 (25508, Santa Cruz). For visualisation purposes, fluorescently conjugated secondary mouse (926-32210, LI-COR) and rabbit (926-32211, LI-COR) antibodies were used and detection was performed using the ODYSSEY CLx (LI-COR®).

### Preparation of 5-FU-resistant patient-derived xenograft

In brief, the tumour was surgically removed from the patient and pieces (2 mm × 2 mm) were engrafted into mice and allowed to grow as described previously and approved under WEHI human ethics (14/15) and animal ethics (2020.032) [11]. Following successful engraftment, the xenografts were treated with 5-FU until tumour regression and/or relapse was observed. This was repeated over multiple cycles until treatment resistance was observed. In some instances, after chemoresistant PDX mice were established, xenografts were treated with a higher concentration of 5-FU as indicated.

### Establishing tumour xenografts in athymic nude mice

Animal experiments were conducted as per La Trobe University animal ethics (AEC14-15). LIM1215 Cas9 (2.5 × 10^6^) and NEDD4 KO cells (2.5 × 10^6^) were harvested in vitro and inoculated subcutaneously into the right flank of 6–8-week-old C57BL/6 female athymic mice. Tumour size was measured using a digital calliper and tumour volume was calculated according to the formula 0.5 (*W*^2^ × *L*), where *W* and *L* are the smallest and largest perpendicular diameters. The mice were randomised into two groups: control group (PBS) and 5-FU (40 mg/kg/twice a week) group for the intraperitoneal injections. Body weight and tumour size were monitored triweekly.

### Cell proliferation assay

Equal number of cells were seeded into 96-well plates. Proliferation was detected by MTS assay at 0, 24, 48 and 72 h time points. At indicated time points, 20 µL of MTS solution (PMS reagent (Sigma Life Science®) in DPBS and CellTiter 96® Aqueous MTS reagent powder (Promega) in DPBS at the ratio of 1:20 was added to each well. The plate was incubated for 1.5 h after the addition of the MTS solution. The absorbance was measured at wavelengths 490 and 630 nm using SpectraMaxM5 multi-mode microplate reader (Molecular Devices).

### Trypan blue assay

Trypan blue exclusion test was used to measure cell viability. Equal numbers of cells were seeded in triplicates in 12-well plates and incubated at 37 °C in 5% CO_2_ for 24 and 48 h. At the indicated time points, supernatant media containing floating dead cells was transferred into fresh tubes and adhered cells were trypsinized using 0.25% Trypsin–EDTA. Trypsinized cells were mixed with the dead cells and centrifuged at 1500×*g* for 5 min. After centrifugation, pellets were resuspended in fresh culture medium and mixed with trypan blue (Santa Cruz, CA, USA) to a ratio of 1:1. Cell counting was performed using a Neubauer haemocytometer (ProSciTech, Townsville, QLD, Australia).

### Clonogenic assay

Cells were seeded in six-well plates at a density of 200 cells/well. The cells were cultured for 15 days, stained with 1% (w/v) crystal violet and the colonies were counted.

### Wound healing assay

Equal number of cells were seeded in 12-well plates and allowed to reach 100% confluency. The monolayer of cells was scratched using a pipette tip. Detached cells were removed by replacing the media with fresh media. Cells were then incubated at 37 °C in 5% CO_2_ for 16 h. The width of the wound was monitored under the microscope at 0 and 16 h post scratch. ImageJ software was used to analyse the wound area.

### Statistical analysis

Statistical analysis was performed using a two-tailed *t*-test and *p*-values of <0.05 were considered significant. In vitro experiments were performed with at least three biological replicates. Error bars in graphical data represented by ±SEM.

## Results

### NEDD4 abundance is low in 5-FU-resistant CRC cells

**Fig. 1:**
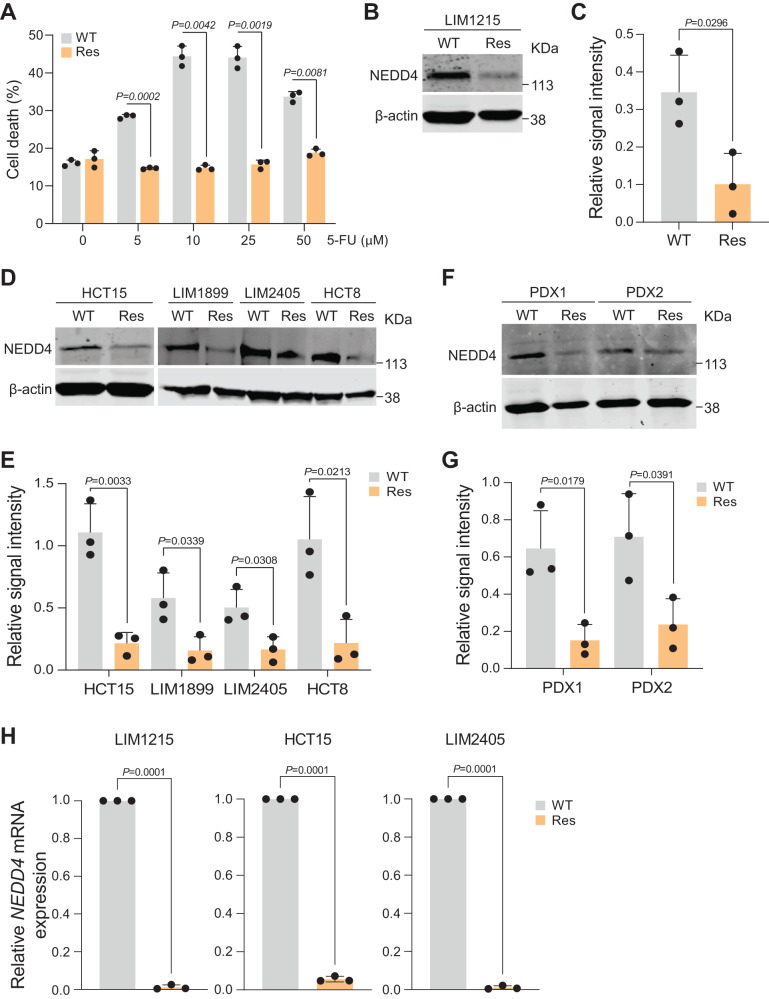
Abundance of NEDD4 is low in CRC cells resistant to 5-FU. **A** Apoptosis of LIM1215 wild-type (WT) and resistant (Res) cells following 5-FU treatment was analysed using flow cytometry (*n* = 3). **B** and **C** Western blot analysis of WT and Res LIM1215 cell lysates for NEDD4 and β-actin. Quantification of NEDD4 intensity normalised to β-actin (*n* = 3). **D** and **E** Western blot analysis of WT and Res CRC cell lysates for NEDD4 and β-actin. Quantification of NEDD4 intensity normalised to β-actin (*n* = 3). **F** and **G** Western blot analysis of WT and Res PDX lysates for NEDD4 and β-actin. Quantification of NEDD4 intensity normalised to β-actin (*n* = 3). **H** Relative mRNA expression of NEDD4 normalised to GAPDH in WT and Res CRC cells is depicted (*n* = 3). All data is represented as mean ± SEM. Significance is determined by two-tailed *t*-test.

To understand the role of NEDD4 in chemoresistance, a panel of 5-FU resistant CRC cells were established by long-time exposure of parental cells with increasing concentrations of 5-FU. Next, a FACS-based apoptosis assay was performed to confirm the resistance of CRC cells (LIM1215) to 5-FU. The apoptosis data confirmed a significant reduction of cell death upon 5-FU treatment in resistant cells compared to parental cells (Fig. 1A). To examine the expression of NEDD4 upon acquiring resistance to 5-FU, Western blot analysis was performed. Western blotting highlighted the low abundance of NEDD4 in 5-FU-resistant LIM1215 CRC cells compared to the parental cells (Fig. 1B, C). To validate this further, a panel of four CRC cell lines with induced resistance to 5-FU were utilised. Consistent with LIM1215-resistant cells, all the CRC cells exhibited low abundance of NEDD4 upon acquiring resistance to 5-FU (Fig. 1D, E). Furthermore, this observation was also confirmed using patient-derived xenograft (PDX) tumour samples that are resistant to 5-FU (Fig. 1F, G). To understand whether NEDD4 is regulated at the transcriptional level, qPCR analysis was conducted on CRC cells. The levels of NEDD4 mRNA were significantly lower in LIM1215, HCT15 and LIM2405 CRC cells that are resistant to 5-FU as compared to the corresponding control cells (Fig. 1H). Taken together, these findings highlight that the E3 ubiquitin ligase NEDD4 is reduced in CRC cells upon acquiring resistance to 5-FU.

### Knockout of NEDD4 protects CRC cells from 5-FU-induced apoptosis

**Fig. 2:**
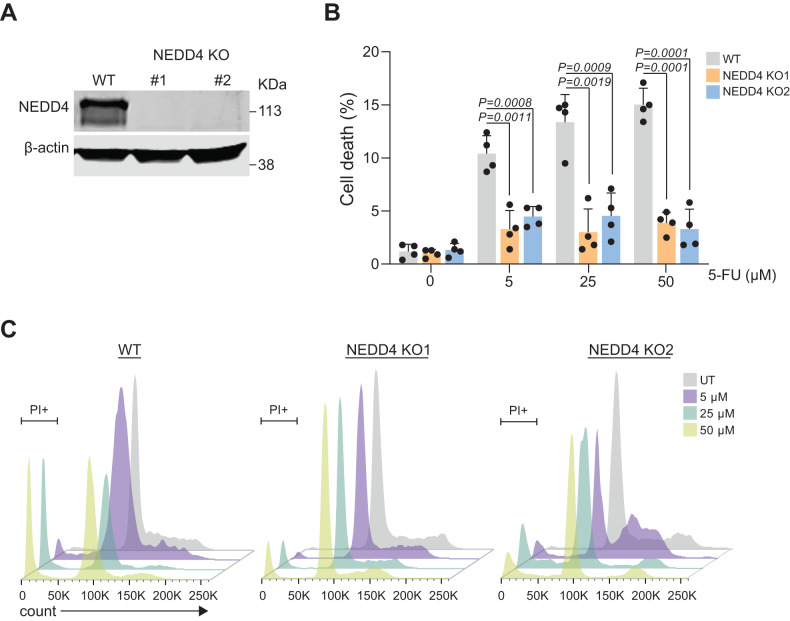
Knockout of NEDD4 protects CRC cells from 5-FU-induced apoptosis. **A** Western blot analysis for the confirmation of NEDD4 KO in LIM1215 cell line. **B** FACS-based apoptosis was performed on LIM1215 WT and *NEDD4* KO treated with 5-FU (5, 25 and 50 µM) for 72 h. Percentage of cell death is represented as percent sub-G1 (*n* = 4). **C** 5-FU (5, 25 and 50 µM) treated WT and *NEDD4* KO LIM1215 cells were analysed for cell cycle profile. All data are represented as mean ± SEM. Significance is determined by two-tailed *t*-test.

Although the abundance of NEDD4 was reduced in 5-FU resistant CRC cells and PDX samples, it was unclear whether NEDD4 has any functional role in conferring resistance to 5-FU. To understand whether the reduction of NEDD4 induces chemoresistance, CRISPR-based LIM1215 *NEDD4* knockout (KO) cells were established. As shown in Fig. 2A, the knockout of NEDD4 in LIM1215 CRC cells was confirmed by Western blotting. To examine 5-FU-induced cell death upon loss of NEDD4, the established CRC cells were treated with increasing concentrations of 5-FU and incubated for 72 h. Interestingly, cell death analysis revealed that *NEDD4* KO LIM1215 cells were protected from 5-FU-induced apoptosis (Fig. 2B). Whilst there was a gradual increase in cell death in WT LIM1215 cells that were incubated with increasing concentrations of 5-FU, this could not be observed in *NEDD4* KO CRC cells. Cell cycle analysis showed no significant difference in the cell cycle profiles between WT and *NEDD4* KO CRC cells. However, in the presence of increasing concentrations of 5-FU, WT cells exhibited more apoptosis than *NEDD4* KO cells (Fig. 2C). To examine whether this phenotype is dependent on the ubiquitination activity of NEDD4, LIM1215 WT and KO cells were transfected with catalytically active and inactive NEDD4 plasmids, treated with 5-FU and subjected to cell death analysis. Expression of WT NEDD4 constructs sensitised the CRC cells to 5-FU but not the catalytically inactive mutants (Supplementary Fig. 1). This observation confirms that 5-FU resistance in CRC cells is dependent upon the E3 ligase activity of NEDD4. Collectively, these data suggest that NEDD4 plays a critical role in the 5-FU response in CRC cells.

### *NEDD4* KO CRC cells do not show cross-resistance against oxaliplatin and irinotecan

**Fig. 3:**
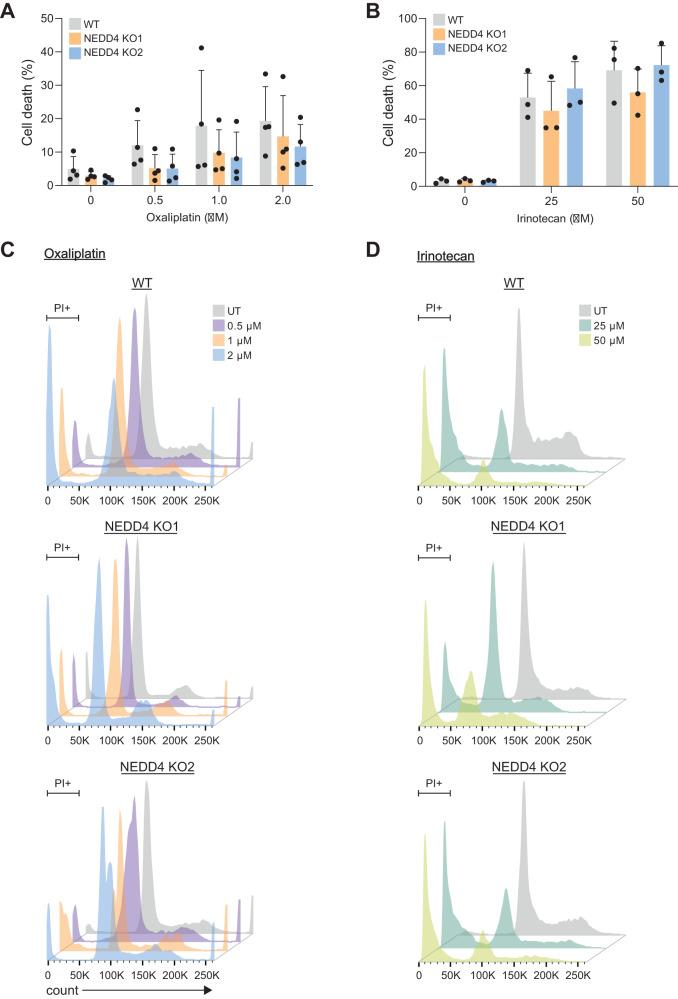
Knockout of NEDD4 does not show cross-resistance to oxaliplatin and irinotecan-induced apoptosis. FACS-based apoptosis was performed on LIM1215 WT and *NEDD4* KO cells treated with or without chemotherapeutic drug **A** oxaliplatin (*n* = 4) (0.5, 1 and 2 µM) and **B** irinotecan (*n* = 3) (25 and 50 µM) for 72 h. Percentage of cell death is represented as percent sub-G1. **C**, **D** Oxaliplatin and irinotecan-treated WT and *NEDD4* KO LIM1215 cells, respectively, were analysed for cell cycle profile. All data are represented as mean ± SEM.

Cells that are resistant to specific chemotherapeutic drugs have the potential to become resistant to other drugs depending on their mode of action. This phenomenon of multidrug resistance has been reported in various animal models and within the clinical setting [12]. As 5-FU is mostly administered in combination with other chemotherapeutic drugs such as oxaliplatin (platinum-based drug) and/or irinotecan (topoisomerase inhibitor), it is important to examine whether loss of NEDD4 protects cells specifically against 5-FU or can confer resistance to other drugs as well [13]. To understand whether loss of NEDD4 desensitises the CRC cells to oxaliplatin and irinotecan, LIM1215 WT and NEDD4 KO cells were subjected to cell death analysis. WT and NEDD4 KO cells were treated with oxaliplatin and irinotecan for 72 h and PI-FACS-based apoptosis assay was performed. As shown in Fig. 3A, NEDD4 KO cells exhibited no significant protection against oxaliplatin. Similarly, irinotecan-treated NEDD4 KO cells did not show a significant difference in apoptosis compared to the WT counterpart (Fig. 3B). In contrast to 5-FU, the cell cycle profiles of NEDD4 KO cells compared to WT cells were not altered upon oxaliplatin or irinotecan treatment (Fig. 3C, D). Together, these data suggest that loss of NEDD4 results in chemoresistance specifically to 5-FU and had no significant impact on the sensitivity to oxaliplatin or irinotecan.

### Loss of NEDD4 alters cellular morphology and reduces cell proliferation

**Fig. 4:**
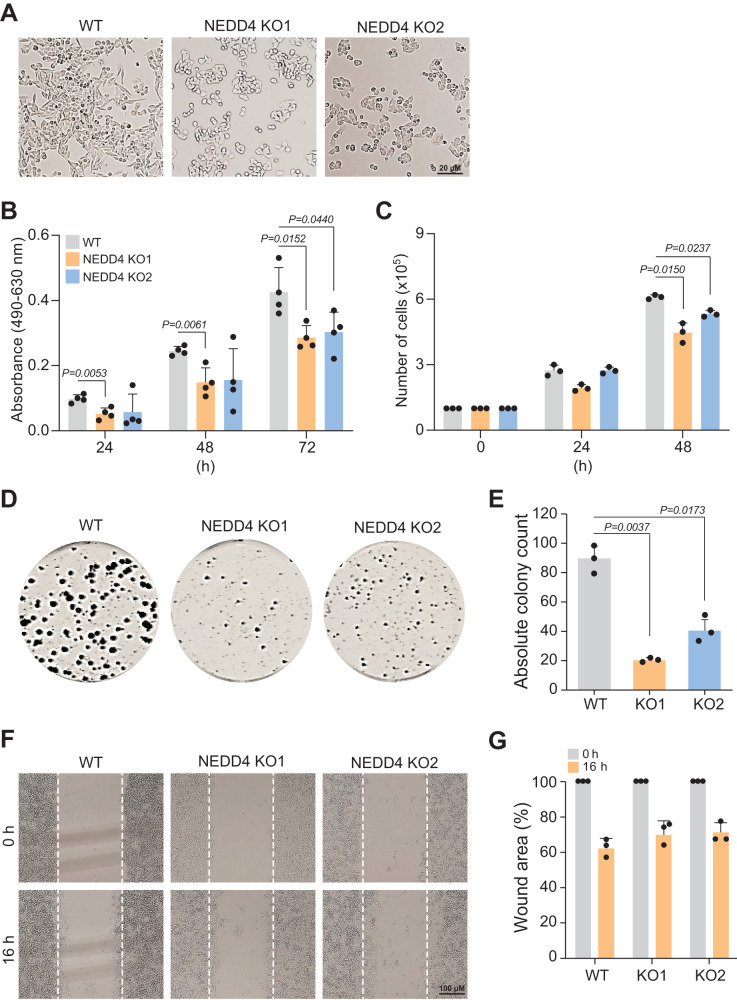
Loss of NEDD4 altered cell morphology and reduced cell proliferation. **A** NEDD4 knockout in CRC cells leads to altered morphology. Representative phase contrast micrographs of LIM1215 WT and *NEDD4* KO cells are shown. **B** Cell proliferation was measured by MTS assay at 24, 48 and 72 h (*n* = 4). **C** Reduced cell growth in *NEDD4* KO clones was analysed using trypan blue assay which was performed at 24 and 48 h (*n* = 3). **D** Defects in two *NEDD4* KO clones were analysed using colony formation assay. **E** Graphical representation of number of colonies counted (*n* = 3). **F** Representative image of wound healing assay LIM1215 WT and *NEDD4* KO cells. The wound was created after the cells reached 100% confluency, migration was assessed at T0 and 16 h post-wounding. Images were taken under the ×4 objective of the light microscope. **G** Quantification of wound closure is showed (*n* = 3). All data are represented as mean ± SEM.

Next, in order to characterise the phenotype of NEDD4 KO cells, we first investigated whether loss of NEDD4 impacted the morphology of LIM1215 cells. Interestingly, small and circular cells were observed upon loss of NEDD4 whereas WT cells were visualised as elongated spindle-shaped (Fig. 4A). Next the role of NEDD4 in cell proliferation was investigated using MTS (Fig. 4B) and trypan blue assay (Fig. 4C). MTS cell proliferation assay indicated that NEDD4 KO cells were significantly less metabolically active compared to the WT counterpart. This observation was further supported by trypan blue assay which showed a significantly slower rate of proliferation at 48 h. Further to this, colony forming assay showed a significant reduction in colony formation in NEDD4 KO LIM1215 cells compared to WT (Fig. 4D, E). However, wound healing assay showed no significant difference in cell migration at 16 h suggesting that NEDD4 does not regulate cellular migration in LIM1215 CRC cells (Fig. 4F, G). Together, these findings indicate that NEDD4 plays an important role in the proliferation and colony formation of CRC cells.

### Loss of NEDD4 does not induce epithelial-to-mesenchymal transition and autophagy

**Fig. 5:**
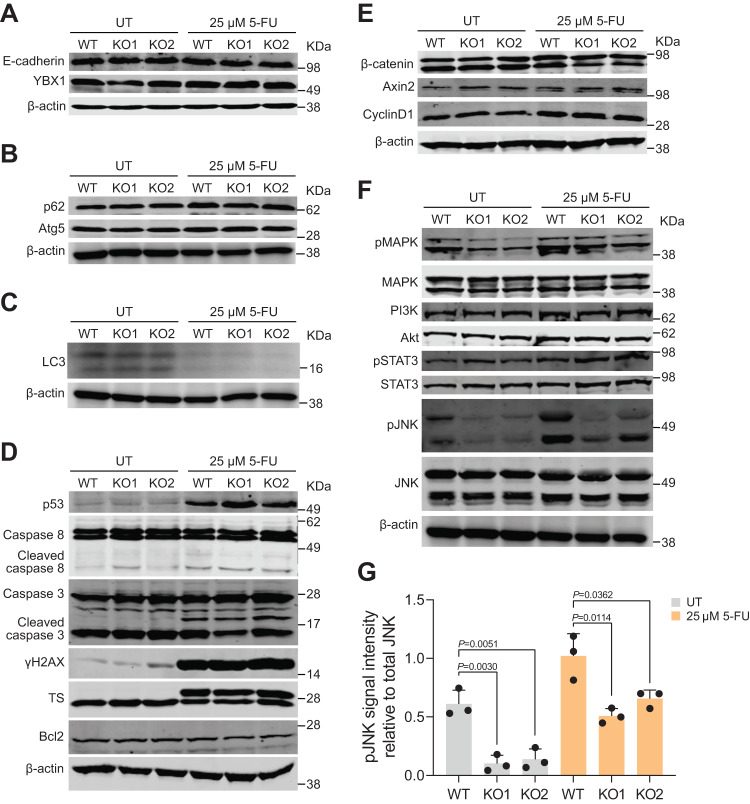
Loss of NEDD4 alters JNK signalling pathway. **A** Western blot analysis of epithelial marker E-cadherin and mesenchymal marker YBX1. **B** Western blot analysis of autophagy markers p62 and Atg5. β-actin was used for a loading control. **C** Western blot analysis of autophagy marker LC3. **D** Western blot analysis of pro-apoptotic markers p53, caspase 3, caspase 8, DNA damage repair marker gamma-H2AX and anti-apoptotic marker BCL2 and enzyme thymidylate synthase (TS) was performed following 5-FU treatment. **E** Western blot analysis of β-catenin and Wnt target genes Axin 2 and Cyclin D1 following 5-FU treatment. **F** Western blot analysis of cellular signalling pathways pMAPK, PI3K/AKT, pSTAT3 and pJNK following 5-FU treatment. **G** Quantification of pJNK intensity normalised to total JNK (*n* = 3). All data is represented as mean ± SEM. Significance is determined by two-tailed *t*-test.

Among the various mechanisms that have been proposed to regulate chemoresistance, epithelial-to-mesenchymal transition (EMT) and autophagy have been studied extensively [14]. EMT is a complex phenomenon where epithelial cells lose polarity and gain mesenchymal attributes. It is characterised by the loss of epithelial markers such as E-cadherin and the gain of mesenchymal markers like Vimentin and YBX1 [14, 15]. To investigate if EMT is linked to the 5-FU chemoresistance due to the loss of NEDD4, Western blotting was performed for E-cadherin and YBX1 using WT and NEDD4 KO cell lysates. However, no significant difference in the expression levels of E-cadherin and YBX1 was observed suggesting that EMT is not induced upon loss of NEDD4 (Fig. 5A—full blot provided in Supplementary Fig. 2).

Next, the role of autophagy was investigated in NEDD4 KO cells. Autophagy is a catabolic process where cellular proteins and organelles are degraded via the lysosomal pathway under adverse conditions such as nutrient deficiency [16]. When autophagy is induced, p62 is degraded and hence p62 is used as an autophagy marker [17, 18]. Apart from p62, Atg5 and LC3 have been shown to regulate autophagy and facilitate the formation of autophagosome [19, 20]. Western blotting confirmed no significant change in the expression level of autophagy marker p62 and autophagy regulators Atg5 and LC3 (Fig. 5B, C). These findings suggest that loss of NEDD4 does not induce EMT and autophagy.

### Expression levels of pro- and anti-apoptotic proteins were not altered in WT and NEDD4 KO cells

Next, the role of pro- and anti-apoptotic proteins in *NEDD4* KO cells were investigated. To do this, cells were treated with 25 µM of 5-FU for 72 h, followed by Western blot analysis. The expression levels of p53, the most widely studied tumour suppressor were examined first. No significant difference in the levels of p53 was observed between WT and *NEDD4* KO cells with or without 5-FU treatment (Fig. 5D). Similarly, treatment with 5-FU did not alter the expression of pro-apoptotic markers such as caspase 3 and caspase 8 in *NEDD4* KO cells compared to WT cells. Furthermore, levels of DNA damage marker gamma-H2AX (γH2AX) was unaltered. As the BCL-2 family protein protects cells from apoptosis [21, 22] and is implicated in chemoresistance [23], the expression of the anti-apoptotic protein BCL-2 was examined. Western blot analysis revealed no marked difference in the expression levels of BCL-2 upon 5-FU treatment in *NEDD4* KO cells. These results suggest that no significant changes could be observed for pro- and anti-apoptotic proteins upon loss of NEDD4. Next, as the protection of apoptosis was specific to 5-FU, specific proteins implicated in 5-FU metabolism were investigated. Thymidylate synthase (TS) is an enzyme that is inhibited by the 5-FU active metabolite, fluorodeoxyuridine monophosphate (FdUMP) thereby causing DNA damage [24]. However, Western blotting analysis showed that the expression level of TS remained unaffected in *NEDD4* KO cells. Taken together, these results suggest that protection against 5-FU in NEDD4 KO cells is not mediated by Caspase 3, Caspase 8, BCL-2 and TS.

### Loss of NEDD4 alters JNK signalling pathway

Next, the role of altered cellular signalling pathways was investigated in *NEDD4* KO CRC cells. To investigate this, genes implicated in Wnt signalling were examined by Western blotting. No significant difference was observed in the expression levels of β-catenin, Axin 2 and Cyclin D1 (Fig. 5E). Similarly, no change could be detected in the expression levels of pMAPK, PI3K/AKT and pSTAT3 in *NEDD4* KO LIM1215 CRC cells (Fig. 5F). However, a marked difference was observed in the expression levels of pJNK in *NEDD4* KO CRC cells, yet expression level of total JNK remained unaltered (Fig. 5F, G). Though, treatment of CRC cells with 5-FU increased the levels of pJNK, the increase in abundance was higher in WT cells relative to NEDD4 KO cells. These data suggest that loss of NEDD4 results in the dysregulation of the JNK signalling pathway.

### Loss of NEDD4 reduces the tumour burden

**Fig. 6:**
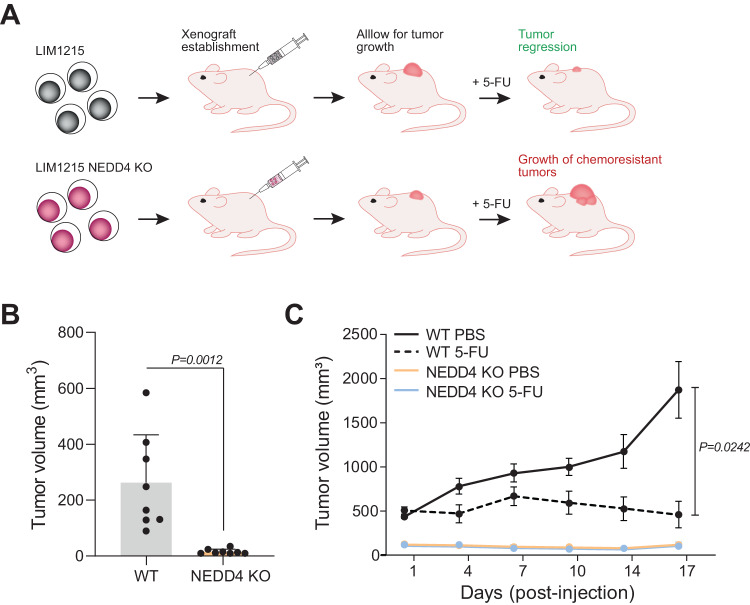
Loss of NEDD4 reduces primary tumour burden. **A** Diagrammatic depiction of the method used for mouse xenograft establishment. Athymic nude mice, at 6–8 weeks of age, received subcutaneous transplantation of either WT or *NEDD4* KO LIM1215 cells. Following tumour growth mice were treated with 5-FU. It was hypothesised that the *NEDD4* KO LIM1215 tumour would be smaller and resistant to 5-FU based on the in vitro data. **B** Tumour volume was measured using a digital calliper post-5-FU treatment (*n* = 8). **C** Tumour volume with or without 5-FU treatment over the course of the experiment (*n* = 8). All data are represented as mean ± SEM.

Next, we sought to determine if *NEDD4* KO cells can regulate tumour growth and chemoresistance in vivo. To do this, 6–8-week-old C57BL/6 athymic nude mice were subcutaneously injected with WT and NEDD4 KO (2.5 × 10^5^) cells and tumour growth was monitored. A schematic representation of the experimental design and expected results are shown in Fig. 6A. Consistent with in vitro data of cell proliferation and colony formation, *NEDD4* KO cells exhibited significantly smaller tumours 17 days post injection (Fig. 6B). The average size of *NEDD4* KO mouse xenografts tumour was < 50 mm^3^ whereas WT xenograft tumour grew up to 250 mm^3^. Next, we investigated if *NEDD4* KO CRC cells are protected from 5-FU-mediated cell death in vivo. For this, we administered 5-FU (40 mg/kg) and PBS intraperitoneally. Mice were monitored for tumour size and body weight post-treatment. As shown in Fig. 6C, 5-FU treatment caused significant regression of the primary tumour in WT CRC cell implanted mice. Mice bearing *NEDD4* KO tumour, though small, exhibited no reduction in tumour burden upon 5-FU treatment. Taken together, these results indicate that loss of NEDD4 reduces tumour growth and altered response to 5-FU.

### Activation of JNK signalling sensitises NEDD4 KO cells to 5-FU

**Fig. 7:**
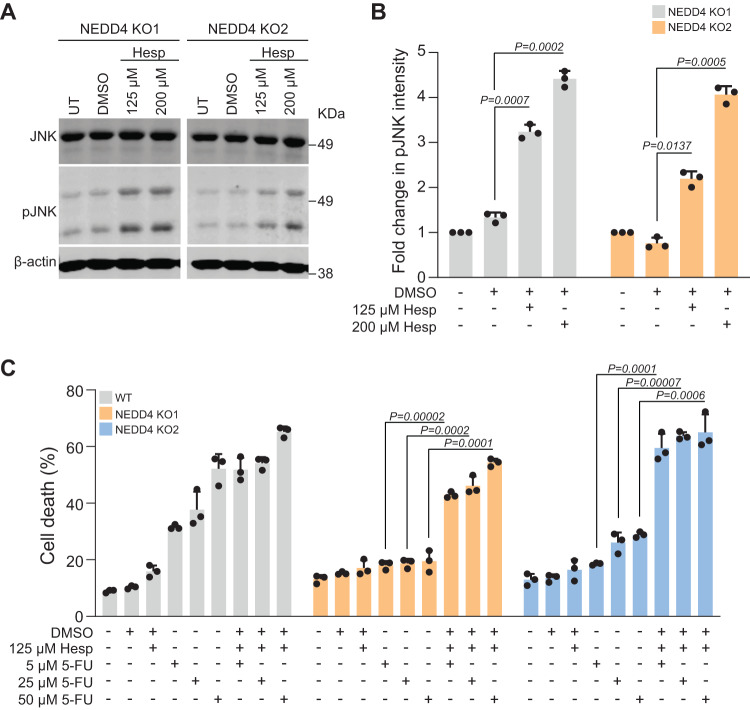
JNK signalling activation sensitises CRC cells to 5-FU. **A** Western blot analysis of pJNK and total JNK following treatment with JNK activator hesperidin of two independent *NEDD4* KO clones in LIM1215 cells. **B** Quantitative representation of pJNK in Western blots (*n* = 3). **C** FACS-based apoptosis was performed on LIM1215 WT and *NEDD4* KO treated with 5-FU (5, 25 and 50 µM) and/or hesperidin (125 µM) for 72 h. Percentage of cell death is represented as percent sub-G1 *(n* = 3). All data are represented as mean ±SEM.

As the JNK signalling pathway was inhibited upon loss of NEDD4, it was hypothesised that activation of JNK signalling could sensitise the NEDD4 KO cells to 5-FU. To test this hypothesis, *NEDD4* KO cells were treated with hesperidin and subjected to Western blotting. As shown in Fig. 7A, B treatment of hesperidin significantly activated the JNK signalling pathway in *NEDD4* KO cells. Next, WT and *NEDD4* KO cells were treated with hesperidin and/or 5-FU and subjected to cell death analysis. As shown in Fig. 7C, treatment of *NEDD4* KO cells with hesperidin increased the sensitivity to 5-FU similar to WT cells. It should be noted that co-treatment of hesperidin and 5-FU also increased cell death in WT CRC cells. These data suggest that NEDD4 loss inhibits JNK signalling which in turn alters the sensitivity of CRC cells to 5-FU.

## Discussion

Recent studies have examined the role of NEDD4 in tumorigenesis amongst several types of cancer. Currently, NEDD4 is suggested to be overexpressed in various cancers including CRC and malignant gastric cancer [25, 26]. However, the pivotal role of NEDD4 as an oncoprotein or tumour suppressor is still debated. For instance, few studies reported NEDD4 as a tumour suppressor while others have reported NEDD4 to promote cancer growth [27]. Considerable reduction in cellular proliferation was reported when NEDD4 was knocked down using siRNA in HCT-15 and LoVo CRC cell lines. In addition, the loss of NEDD4 also impaired cell morphology in both cell lines [28]. Similarly, NEDD4 has been implicated in the progression of non-small cell lung carcinoma (NSCLC). Overexpression of NEDD4 resulted in PTEN ubiquitination, decreased PTEN stability and reduced the proliferation of NSCLC cells [29]. Furthermore, similar observations were reported in pancreatic adenocarcinoma (PDAC) cell lines where NEDD4-regulated PTEN expression negatively and led to uncontrolled cell growth and metastasis of pancreatic cancer [30]. This accumulating evidence suggested that E3 ligase NEDD4 is a potential target for cancer therapeutics. In contrast, Lu et al. found that depletion of NEDD4 has no impact on tumorigenesis by itself however loss of NEDD4 in a sporadic CRC model resulted in enhanced tumour growth in the context of Apc^+/min^ associated cancer [8]. In addition, NEDD4 has also been implicated in chemoresistance in nasopharyngeal carcinoma (NPC). NEDD4 was shown to regulate cisplatin resistance of NPC cells by inducing EMT [7]. Similarly, NEDD4 has been implicated to induce chemosensitivity in lung adenocarcinoma cells through inhibition of PTEN [6]. Likewise, overexpression of NEDD4 is responsible for afatinib resistance of NSCLC cells [31]. NEDD4 has been shown to regulate MDM2 ubiquitination and thereby regulate p53 activity in the cells [32]. As loss of NEDD4 increased P53 activity and increased the DNA damage response, it can be speculated that NEDD4 can regulate chemoresistance in a P53-dependent manner.

In this study, we demonstrate that the loss of NEDD4 in CRC cells confers a 5-FU-resistant phenotype. Loss of NEDD4 altered colony-forming abilities and reduced the proliferation of the CRC cells. Though the reduced proliferation can affect the sensitivity of the cells to chemotherapeutic drugs, *NEDD4* KO cells were resistant only to 5-FU and other chemotherapeutic drugs such as irinotecan and oxaliplatin were able to induce apoptosis despite reduced proliferation. These results suggest that other mechanisms exist that control chemoresistance to 5-FU in *NEDD4* KO cells.

In contrast to previous studies where NEDD4 controlled cisplatin resistance by altering EMT [7], *NEDD4* KO did not induce EMT in LIM1215 CRC cells. In addition, no change in autophagy marker p62 was observed upon *NEDD4* KO. This observation was contrary to what is reported in the literature for prostate cancer cell DU145, where NEDD4 knockdown induced autophagy [33]. These results suggest that the regulation of various cellular pathways by NEDD4 could be cell-type dependent. Alternatively, knockdown and knockout of proteins could also render the cell to respond differently. Further analysis of other cell types with CRISPR/Cas9 and RNAi methods is needed to understand the functional role of NEDD4 and the effect of gene knockdown/knockout methods on the phenotype of differing cell types.

Even though the loss of NEDD4 exhibited resistance to 5-FU, a significant reduction in tumour size was also observed upon loss of NEDD4. This finding of chemoresistance and reduced tumour burden also challenges the use of xenograft models to determine whether a protein is a tumour suppressor or a tumour promoter. Historically, after a gene knockout or knockdown, reduced tumour volume in vivo would characterise the lost/silenced protein as a tumour promoter, however, the effect of depletion of the protein in the context of chemotherapy and associated resistance is often overlooked. It was established that *NEDD4* KO did not cause tumour growth sporadically [8], however, when *NEDD4* KO mice were crossed with sporadic CRC models, the tumour burden was significantly accelerated.

As demonstrated in this study, JNK signalling was inhibited in *NEDD4* KO cells. Recent studies have shown the pivotal role of JNK signalling in inherent cisplatin resistance [34]. It is suggested that JNK signalling can activate apoptosis as well as increase resistance to cisplatin‐based chemotherapy in a context-dependent manner. In accordance with this hypothesis, it was previously reported that inhibition of JNK signalling using CC-401 sensitised the CRC cells to various drugs including oxaliplatin, SN-38 and 5-FU [35]. As the context of the study was on the synergistic effect of JNK inhibitor CC-401 and chemotherapeutic drug, it differs significantly from our study of acquired resistance to 5-FU. Further studies are needed to understand the role of JNK signalling in chemotherapy-induced apoptosis and acquired resistance. Nevertheless, in this study, we show that activation of JNK signalling rendered the *NEDD4* KO CRC cells sensitive to 5-FU.

### Reporting summary

Further information on research design is available in the Nature Research Reporting Summary linked to this article.

### Supplementary information

Suplementary Figure

Reporting summary

## Data Availability

All datasets generated and analysed during this study are included in this published article and its Supplementary Information files. Additional data are available from the corresponding author on reasonable request.

## References

[1] He J, Pei L, Jiang H, Yang W, Chen J, Liang H (2017). Chemoresistance of colorectal cancer to 5-fluorouracil is associated with silencing of the BNIP3 gene through aberrant methylation. J Cancer.

[2] Douillard JY, Cunningham D, Roth AD, Navarro M, James RD, Karasek P (2000). Irinotecan combined with fluorouracil compared with fluorouracil alone as first-line treatment for metastatic colorectal cancer: a multicentre randomised trial. The Lancet.

[3] Chocry M, Leloup L, Kovacic H (2017). Reversion of resistance to oxaliplatin by inhibition of p38 MAPK in colorectal cancer cell lines: involvement of the calpain/Nox1 pathway. Oncotarget.

[4] Szaryńska M, Olejniczak A, Kobiela J, Spychalski P, Kmieć Z (2017). Therapeutic strategies against cancer stem cells in human colorectal cancer. Oncol Lett.

[5] Callis J (2014). The ubiquitination machinery of the ubiquitin system. Arabidopsis Book/Am Soc Plant Biol.

[6] Song Y-H, Zhang C-Q, Chen F-F, Lin X-Y (2018). Upregulation of neural precursor cell expressed developmentally downregulated 4-1 is associated with poor prognosis and chemoresistance in lung adenocarcinoma. Chin Med J.

[7] Feng S, Yang G, Yang H, Liang Z, Zhang R, Fan Y (2017). NEDD4 is involved in acquisition of epithelial–mesenchymal transition in cisplatin-resistant nasopharyngeal carcinoma cells. Cell Cycle.

[8] Lu C, Thoeni C, Connor A, Kawabe H, Gallinger S, Rotin D (2016). Intestinal knockout of Nedd4 enhances growth of Apcmin tumors. Oncogene.

[9] Sun A, Yu G, Dou X, Yan X, Yang W, Lin Q (2014). Nedd4-1 is an exceptional prognostic biomarker for gastric cardia adenocarcinoma and functionally associated with metastasis. Mol Cancer.

[10] Wang ZW, Hu X, Ye M, Lin M, Chu M, Shen X (2020). NEDD4 E3 ligase: Functions and mechanism in human cancer. Semin Cancer Biol.

[11] Abdirahman SM, Christie M, Preaudet A, Burstroem MCU, Mouradov D, Lee B (2020). A biobank of colorectal cancer patient-derived xenografts. Cancers (Basel).

[12] Giaccone G, Pinedo HM (1996). Drug resistance. Oncologist.

[13] Calvo E, Cortés J, González-Cao M, Rodríguez J, Aramendía JM, Fernández-Hidalgo Ó (2002). Combined irinotecan, oxaliplatin and 5-fluorouracil in patients with advanced colorectal cancer. Oncology.

[14] Gatti L, Zunino F (2005). Overview of tumor cell chemoresistance mechanisms. Methods Mol Med.

[15] Housman G, Byler S, Heerboth S, Lapinska K, Longacre M, Snyder N (2014). Drug resistance in cancer: an overview. Cancers.

[16] Holohan C, Van Schaeybroeck S, Longley DB, Johnston PG (2013). Cancer drug resistance: an evolving paradigm. Nat Rev Cancer.

[17] Villarroya-Beltri C, Baixauli F, Mittelbrunn M, Fernández-Delgado I, Torralba D, Moreno-Gonzalo O (2016). ISGylation controls exosome secretion by promoting lysosomal degradation of MVB proteins. Nat Commun.

[18] Zhang P, Lai Z-L, Chen H-F, Zhang M, Wang A, Jia T (2017). Curcumin synergizes with 5-fluorouracil by impairing AMPK/ULK1-dependent autophagy, AKT activity and enhancing apoptosis in colon cancer cells with tumor growth inhibition in xenograft mice. J Exp Clin Cancer Res.

[19] Codogno P, Meijer AJ (2006). Atg5: more than an autophagy factor. Nat Cell Biol.

[20] Pyo J-O, Yoo S-M, Ahn H-H, Nah J, Hong S-H, Kam T-I (2013). Overexpression of Atg5 in mice activates autophagy and extends lifespan. Nat Commun.

[21] Tsujimoto Y (1998). Role of Bcl‐2 family proteins in apoptosis: apoptosomes or mitochondria?. Genes Cells.

[22] Willis S, Day CL, Hinds MG, Huang DCS (2003). The Bcl-2-regulated apoptotic pathway. J Cell Sci.

[23] Reed JC, Kitada S, Takayama S, Miyashita T (1994). Regulation of chemoresistance by the bcl-2 oncoprotein in non-Hodgkin’s lymphoma and lymphocytic leukemia cell lines. Ann Oncol.

[24] Longley DB, Harkin DP, Johnston PG (2003). 5-Fluorouracil: mechanisms of action and clinical strategies. Nat Rev Cancer.

[25] Tanksley JP, Chen X, Coffey RJ (2013). NEDD4L is downregulated in colorectal cancer and inhibits canonical WNT signaling. PLoS ONE.

[26] Kim SS, Yoo NJ, Jeong EG, Kim MS, Lee SH (2008). Expression of NEDD-1, a PTEN regulator, in gastric and colorectal carcinomas. APMIS.

[27] Huang X, Chen J, Cao W, Yang L, Chen Q, He J (2019). The many substrates and functions of NEDD4-1. Cell Death Dis.

[28] Eide PW, Cekaite L, Danielsen SA, Eilertsen IA, Kjenseth A, Fykerud TA (2013). NEDD4 is overexpressed in colorectal cancer and promotes colonic cell growth independently of the PI3K/PTEN/AKT pathway. Cell Signal.

[29] Amodio N, Scrima M, Palaia L, Salman AN, Quintiero A, Franco R (2010). Oncogenic role of the E3 ubiquitin ligase NEDD4-1, a PTEN negative regulator, in non-small-cell lung carcinomas. Am J Pathol.

[30] Weng M, Luo Z-L, Wu X-L, Zeng W-Z (2017). The E3 ubiquitin ligase NEDD4 is translationally upregulated and facilitates pancreatic cancer. Oncotarget.

[31] Booth L, Roberts JL, Poklepovic A, Dent P (2017). NEDD4 over-expression regulates the afatinib resistant phenotype of NSCLC cells. Oncol Signal.

[32] Xu C, Fan CD, Wang X (2015). Regulation of Mdm2 protein stability and the p53 response by NEDD4-1 E3 ligase. Oncogene.

[33] Li Y, Zhang L, Zhou J, Luo S, Huang R, Zhao C (2015). Nedd4 E3 ubiquitin ligase promotes cell proliferation and autophagy. Cell Prolif.

[34] Yan D, An G, Kuo MT (2016). C‐Jun N‐terminal kinase signalling pathway in response to cisplatin. J Cell Mol Med.

[35] Vasilevskaya IA, Selvakumaran M, Hierro LC, Goldstein SR, Winkler JD, O’Dwyer PJ (2015). Inhibition of JNK sensitizes hypoxic colon cancer cells to DNA-damaging agents. Clin Cancer Res.

