# Supplementary material for: The E3 ubiquitin ligase NEDD4 regulates chemoresistance to 5-fluorouracil in colorectal cancer cells by altering JNK signalling
Source: Cell Death Dis. 2023 Dec 14;14(12):828. doi: 10.1038/s41419-023-06349-z (PMC10721789; doi:10.1038/s41419-023-06349-z)

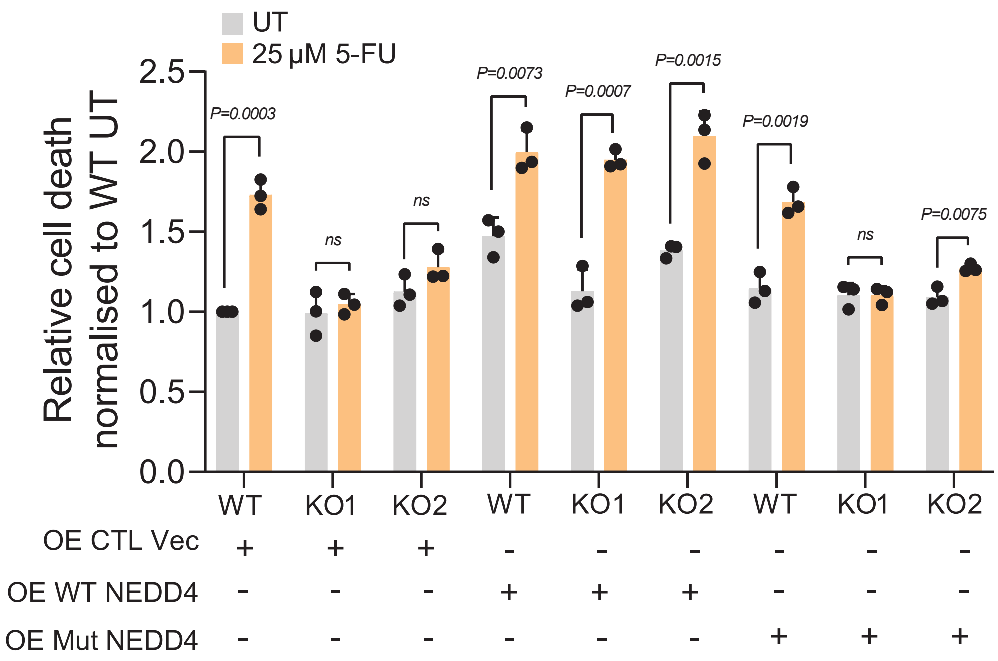

Supplementary Figure 1

Overexpression of WT NEDD4 but not the catalyticaly inactive mutant rescued the cell death phenotype

Cell death assay for WT and NEDD4 KO treated with or without 5-FU in the presence of WT NEDD4 or the catalytically inactive NEDD4 mutant is displayed. Percentage of cell death is represented as percent sub-G1 (n=3). All data are represented as mean ±SEM.

ORIGINAL DATA FILES

Figure 1B

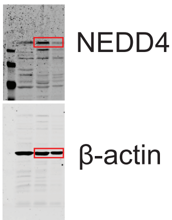

Figure 1D

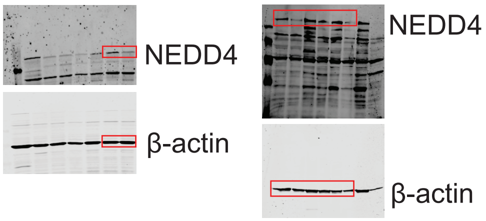

Figure 1F

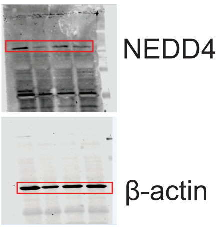

Figure 2A

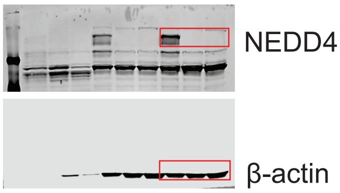

Figure 5A

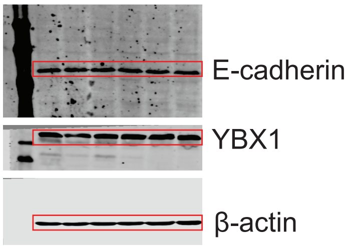

Figure 5B

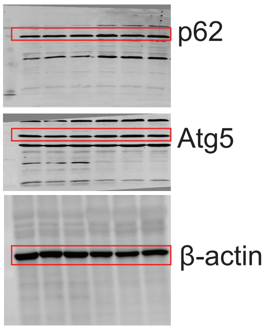

Figure 5C

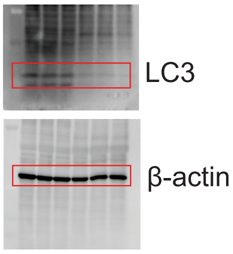

Figure 5D

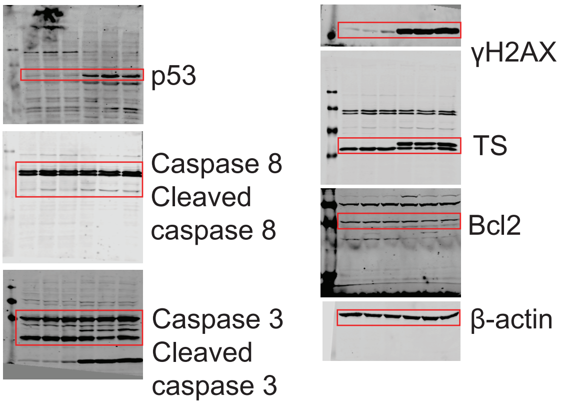

Figure 5E

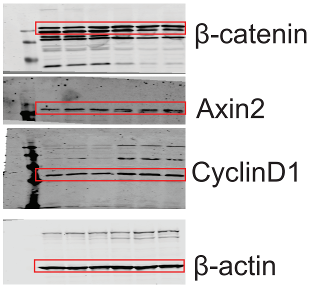

Figure 5F

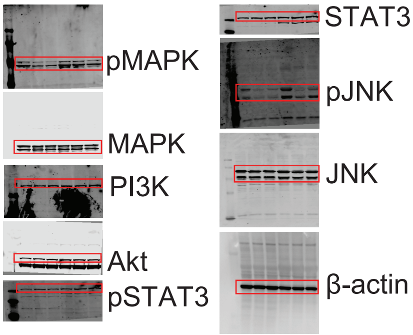

Figure 7A

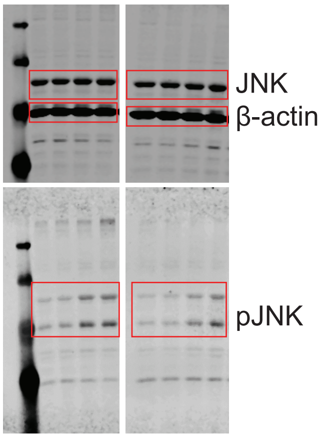

Supplement: Supplementary file 1 — Suplementary Figure [file 41419_2023_6349_MOESM1_ESM.pdf]
